# Inferring Continuous and Discrete Population Genetic Structure Across Space

**DOI:** 10.1534/genetics.118.301333

**Published:** 2018-07-19

**Authors:** Gideon S. Bradburd, Graham M. Coop, Peter L. Ralph

**Affiliations:** * Ecology, Evolutionary Biology, and Behavior Graduate Group, Department of Integrative Biology, Michigan State University, East Lansing, Michigan 48824; † Center for Population Biology, Department of Evolution and Ecology, University of California, Davis, California 95616; ‡ Institute of Ecology and Evolution, Departments of Mathematics and Biology, University of Oregon, Eugene, Oregon 97403

**Keywords:** population genetics, isolation by distance, population structure, model-based clustering

## Abstract

A classic problem in population genetics is the characterization of discrete population structure in the presence of continuous patterns of genetic differentiation. Especially when sampling is discontinuous, the use of clustering or assignment methods may incorrectly ascribe differentiation due to continuous processes (*e.g.*, geographic isolation by distance) to discrete processes, such as geographic, ecological, or reproductive barriers between populations. This reflects a shortcoming of current methods for inferring and visualizing population structure when applied to genetic data deriving from geographically distributed populations. Here, we present a statistical framework for the simultaneous inference of continuous and discrete patterns of population structure. The method estimates ancestry proportions for each sample from a set of two-dimensional population layers, and, within each layer, estimates a rate at which relatedness decays with distance. This thereby explicitly addresses the “clines versus clusters” problem in modeling population genetic variation, and remedies some of the overfitting to which nonspatial models are prone. The method produces useful descriptions of structure in genetic relatedness in situations where separated, geographically distributed populations interact, as after a range expansion or secondary contact. We demonstrate the utility of this approach using simulations and by applying it to empirical datasets of poplars and black bears in North America.

## Abstract (precis)

An important step in the analysis of genetic data is to describe and categorize natural variation. Individuals that live close together are, on average, more genetically similar than individuals sampled farther apart...

A fundamental quandary in the description of biological diversity is the fact that diversity shows both discrete and continuous patterns. For example, reasonable people can disagree about whether two populations are separate species because the process of speciation is usually gradual, and so there is no set point in the continuous divergence of populations when they unambiguously become distinct species. The issue of identifying meaningful biological subunits extends below the species level, as patterns of phenotypic and genetic diversity within and among populations are shaped by continuous migration and drift, as well as by more discrete events, such as rapid expansions, bottlenecks, rare long-distance migration, and separation by geographic barriers. Both discrete and continuous components are required to accurately describe most species’ patterns of genetic relatedness.

From a practical standpoint, we often need to identify somewhat separable populations from which individuals are sampled (Wright 1949), even while acknowledging continuous processes. Delineating populations is useful for systematics and for informing conservation priorities (Moritz 1994; Waples 1998; Moritz *et al.* 2002). Furthermore, we often need to identify subsets of individuals resulting from reasonably coherent evolutionary histories for downstream analyses to learn about population history and adaptation. Conversely, the substantial information available from continuous, geographic differentiation (*e.g.*, adaptation along a climatic gradient) can be confounded by discrete historical processes (*e.g.*, admixture), requiring methods that can disentangle the two.

There have been many methods proposed to characterize population genetic structure, including generating population phylogenies (Cavalli-Sforza and Piazza 1975; Pickrell and Pritchard 2012), dimensionality-reduction approaches such as principal components analysis (Menozzi *et al.* 1978; Price *et al.* 2006; Novembre and Stephens 2008; Meirmans 2009), and model-based clustering approaches (*e.g.*, Pritchard *et al.* 2000; Corander *et al.* 2003; Falush *et al.* 2003; Guillot *et al.* 2005; Huelsenbeck and Andolfatto 2007; Alexander *et al.* 2009; Hubisz *et al.* 2009; Lawson *et al.* 2012; Raj *et al.* 2014; Caye *et al.* 2018). Each of these methods performs best in particular situations, but many can give misleading results when applied to data that show a continuous pattern of differentiation, as that produced by geographic isolation by distance (Wright 1943; Novembre and Stephens 2008; Frantz *et al.* 2009). Here, we will focus on model-based clustering, the most widely used class of approaches for population delineation. (We note that the problem of identifying population clusters is distinct from, though of course related to, the problem of detecting barriers to gene flow between populations, (*e.g.*, Barton 2008; Bradburd *et al.* 2013; Petkova *et al.* 2016; Ringbauer *et al.* 2018). Existing model-based clustering methods model each individual’s genotypes as random draws from a set of underlying, unobserved population clusters, each with a characteristic set of allele frequencies, which are estimated. These underlying frequencies are identical for all individuals assigned to a cluster, regardless of their spatial location. Spatial information has been incorporated into some of these methods, by, for example, placing spatial priors on cluster membership (Guillot *et al.* 2005; Caye *et al.* 2018), but this does not address the underlying issue that these methods assume that allele frequencies are constant in a cluster across the species’ range.

Isolation by distance refers to a pattern of increasing genetic differentiation with geographic separation, which occurs when geographically restricted dispersal allows genetic drift to build up differentiation between distant locations (Wright 1943). Theoretical work, mostly derived from “stepping-stone” models (Kimura and Weiss 1964; Sawyer 1976; Shiga 1988), gives us some analytical predictions for isolation by distance (Malécot 1969; Slatkin 1985; Epperson 2003), and some theory has been derived for continuous space (Nagylaki 1978; Nagylaki and Barcilon 1988; Barton *et al.* 2002), but substantial work remains to be done (Barton *et al.* 2013). Given the generality of the circumstances that generate a pattern of isolation by distance, it is unsurprising that isolation by distance is very widespread in nature (Meirmans 2012; Sexton *et al.* 2014).

The ubiquity of isolation by distance presents a challenge for models of discrete population structure, as it is frequently difficult to determine whether observed patterns of genetic variation are continuously distributed across a landscape, or instead are partitioned in discrete clusters. This problem can be compounded if sampling is done unevenly or discretely across a population or species’ range, and has given rise to a debate in the population genetic literature about how best to describe sets of individuals using continuous clines and discrete clusters (*e.g.*, Serre and Pääbo 2004; Rosenberg *et al.* 2005).

Most existing model-based clustering methods are based on a discrete set of clusters, and so tend to partition continuous variation into spurious clusters with spatially autocorrelated cluster membership (Frantz *et al.* 2009; Meirmans 2012). In analyses of empirical datasets, which often show strong isolation by distance, model-based clustering approaches will therefore tend to overestimate the number of discrete clusters present.

To address this, we set out to develop a model-based clustering method that, when possible, uses isolation by distance to explain observed genetic variation. With an explicit spatial component, discrete population structure need only be invoked when genetic differentiation in the data deviates significantly from that expected given geographic separation. In this paper, we model genetic variation in genotyped individuals as partitioned within or admixed across a specified number of discrete layers, within each of which relatedness decays as a parametric function of the distance between samples. We also implement a cross-validation approach for comparing and selecting models across different numbers of layers, and we demonstrate the utility of our approach using both simulated and empirical data. The implementation of this method, conStruct (for “*con*tinuous *struct*ure”), is documented and available for general use as an R package at https://github.com/gbradburd/conStruct.

## Materials and Methods

### Data

The statistical framework of our approach is conceptually similar to that in Wasser *et al.* (2004), Bradburd *et al.* (2013), and Bradburd *et al.* (2016), although we use a somewhat different summary statistic than in this previous work. The model works with allele frequencies at *L* unlinked, biallelic single nucleotide polymorphisms (SNPs) genotyped across *N* samples. Each “sample” may be a single individual, a collection of individuals from a location, or frequencies estimated from pooled sequencing. From these we compute the *allelic covariance* between samples *i* and *j*, denoted ${\hat{\Omega }}_{i,j},$ as the expected covariance of distinct individual alleles chosen from each of the two samples at a random locus. More precisely, suppose that we pick a random biallelic locus uniformly from the genome, pick a random “reference” allelic state from the two alleles seen at that locus, and, in each sample, draw one random allele, recording $X_{i}=1$ if the allele drawn in sample *i* matches the random reference, and $X_{i}=0$ otherwise. Then,

$${\hat{\Omega }}_{i,j}=\mathrm{cov}\left[X_{i},X_{j}\right].$$ (1)

Because we randomly choose the reference allele, each $X_{i}$ behaves marginally as a fair coin—in particular, $\mathbb{P}\left\{X_{i}=1\right\}=1/2,$ so ${\hat{\Omega }}_{i,i}=1/4$ for every *i*—all information enters through *correlations*.

Although we describe this as a covariance between individually drawn alleles, ${\hat{\Omega }}_{i,j}$ is in fact also the covariance between the allele frequencies of a randomly chosen allele in samples *i* and *j*, as long as i≠j. The choice of allele does not affect subsequent calculations, and so may be arbitrary, and $\hat{\Omega }$ can be calculated as (derived in *Allelic covariance and inference*):

$${\hat{\Omega }}_{i,j}=\frac{1}{L}\sum_{\ell =1}^{L}\left(f_{i,\ell }-1/2\right)\left(f_{j,\ell }-1/2\right)\;\;\text{for }i\neq j.$$ (2)

Here $f_{i,\ell }$ is the allele frequency in the $i^{\text{th}}$ sample at locus ℓ. This definition of covariance differs from the usual “genetic covariance” (McVean 2009) in that (a) we do not subtract locus means (to make the statistic insensitive to sample configuration), and (b) we randomly choose a reference allele at each locus (to retain insensitivity to choice of reference allele). As noted in Petkova *et al.* (2016), for i≠j, this can also be calculated as $\Omega_{i,j}=\left(1-2\pi_{i,j}\right)/4,$ where $\pi_{i,j}$ is the genetic distance calculated from those *L* sites, *i.e.*, the proportion of sites at which random samples from *i* and *j* differ.

### Continuous and discrete differentiation

**Figure 1:**
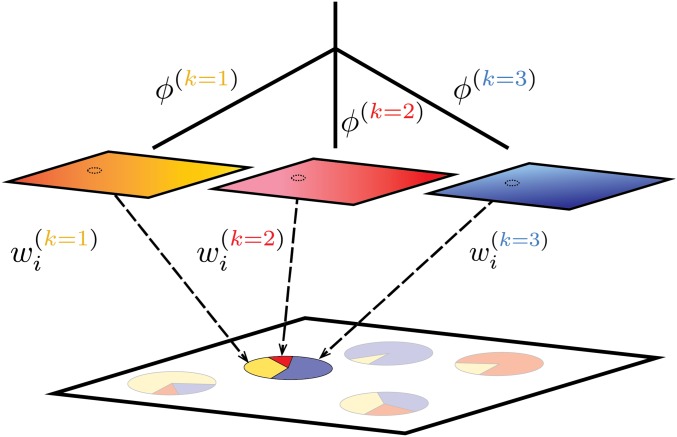
Schematic of our method, using K=3 as an example. Spatial autocorrelation of allele frequencies within each layer is depicted by color gradients, and $\varphi^{\left(k\right)}$ denotes the covariance shared by samples with ancestry entirely in the *k*th layer. Sampled populations on the landscape are inferred to be admixed between these layers; the *i*th sample draws proportion $w_{i}^{\left(k\right)}$ of its ancestry from layer *k*. For convenience, each layer is depicted as a small square, but in fact, each layer exists everywhere in the sampled area, so the small dashed circles on each layer show where the location of the highlighted admixed sample intersects each layer.

Clustering approaches to describing genetic variation are useful because population history can often be meaningfully described on a coarse scale by interactions between discrete “populations” whose relationships are delimited by patterns of glaciation, large-scale migration, mountain ranges, and the like. Here we add a spatial component within each such discrete historical component, which we refer to as a set of “layers” that overlay the modern map. We imagine each layer as a geographically distributed population that extends over the entire sampled range of the populations. As depicted in Figure 1, each sample is composed of a mixture of contributions from each of these layers, with the relative contributions of each layer described by a set of “admixture proportions” (the $w_{i}^{\left(k\right)}$). These layers thus take the place of “clusters” in clustering methods, but we do not adopt this term, as “spatial cluster” suggests a clustering in space, while our layers may contribute to genetic variation across the entire geographic range.

Within each of these layers, allele frequencies have positive covariance at geographically close locations, but this covariance is allowed to decay as geographic distance increases. This pattern of spatial decay reflects how migration between nearby spatial regions homogenizes allele frequency changes that arise locally due to drift, but less effectively homogenizes geographically distant regions, resulting in a continuous pattern of isolation by distance within each layer. There is a fixed amount of covariance between layers, irrespective of spatial location. Within each layer, allele frequencies are expected to change gradually with distance, but observed frequencies can change abruptly at many loci if the proportions of ancestry individuals derive from each layer (the admixture proportions) do so as well.

To allow flexibility in the form of the decay of allelic covariance with geographic distance within each layer, we define the covariance within layer *k* between samples *i* and *j* to be:

$$G_{i,j}^{\left(k\right)}=\alpha_{0}^{\left(k\right)}\left(\exp\left(-{\left(\alpha_{D}^{\left(k\right)}D_{i,j}\right)}^{\alpha_{2}^{\left(k\right)}}\right)\right)+\varphi^{\left(k\right)}$$ (3)

where the superscript (k) denotes parameters specific to the *k*th layer. The quantity $D_{i,j}$ is the observed geographic distance between samples *i* and *j*, and the $\alpha^{\left(k\right)}$ parameters control the shape of the decay of covariance with distance in the layer. Our choice of a powered-exponential decay, as parameterized by the *α*s, is a flexible and standard choice in spatial statistics (Diggle *et al.* 1998), and is not chosen to match a particular population genetics model. The $\varphi^{\left(k\right)}$ is a parameter that describes the background covariance within the layer. If two samples draw 100% of their ancestry from layer *k*, then their covariance under the model is $G_{i,j}^{\left(k\right)};$ if they are furthermore geographically very close ($D_{i,j}=0$) they will have covariance $\alpha_{0}^{\left(k\right)}+\varphi^{\left(k\right)}.$ If the geographic distance between them is very large, their covariance will be equal to the background level $\varphi^{\left(k\right)}$ within the layer. The “shared drift” parameter $\varphi^{\left(k\right)}$ is analogous to the branch length connecting the *k*th population to the population ancestral to all modeled layers (see, for example, Patterson *et al.* 2012; Peter 2016), although they cannot be directly compared because we are modeling the allelic, rather than genetic, covariance. In “Model rationale: drift, admixture, and space” we lay out a simple model of allele frequencies underlying this covariance model.

We then allow samples to draw their ancestry from more than one layer. The admixture proportion of the *i*th sample in the *k*th layer, denoted $w_{i}^{\left(k\right)},$ gives the genome-wide proportion of alleles from sample *i* that derive from layer *k* (and so $\sum_{k=1}^{K}w_{i}^{\left(k\right)}=1$). A visual representation of the method is shown in Figure 1.

We can then describe the covariance between samples *i* and *j* across all *K* layers, $\Omega_{i,j},$ by summing their within-layer spatial covariances ($G_{i,j}^{\left(k\right)}$ in layer *k*), weighted by the relevant admixture proportions.

$$\Omega_{i,j}=\gamma +\sum_{k=1}^{K}w_{i}^{\left(k\right)}w_{j}^{\left(k\right)}G_{i,j}^{\left(k\right)}+\delta_{i,j}\eta_{i}.$$ (4)

In this equation, $w_{i}^{\left(k\right)}w_{j}^{\left(k\right)}$ is the proportion of alleles that *both* sample *i* and sample *j* have inherited from layer *k*.

In addition to the admixture-weighted sum of the within-layer spatial covariances, this function contains two terms, γ and $\delta_{i,j}\eta_{i}.$ The first, γ, describes the global allelic covariance between all samples, and arises because all samples share an ancestral mean allele frequency at each locus, which generates a base-line covariance. In the final term, $\delta_{i,j}$ is an indicator variable that takes a value of 1 when *i* equals *j* and 0 otherwise, and $\eta_{i}$ adds variance specific to sample *i*. This term on the diagonal of the parametric covariance matrix captures processes shaping variance within the sampled deme, such as inbreeding and the sampling process.

### Likelihood and inference

If the allele frequency deviations at each locus were independent between loci and multivariate normally distributed across populations, their allelic covariance $\hat{\Omega }$ would be Wishart distributed with degrees of freedom equal to *L*, the number of loci genotyped. We use this as a convenient approximation to the true distribution described above, and so define the likelihood of the allelic covariance to be

$$P\left(\hat{\Omega }|\Omega \right)=W\left(L\hat{\Omega }|\Omega ,L\right),$$ (5)

where W is the Wishart likelihood function. Statistical nonindependence between loci (linkage disequilibrium, LD) will decrease the effective number of degrees of freedom. One possible solution, which we have not yet found necessary to implement, would be to estimate an *effective* number of loci by introducing a parameter to modify the given degrees of freedom and thereby informally model linkage between loci (*e.g.*, Petkova *et al.* 2016).

We estimate the values of the parameters of the model using a Bayesian approach. Acknowledging the dependence of the parametric covariance matrix Ω on its constituent parameters w,α,φ,η,γ and on the (observed) geographic distances *D* with the notation Ω(w,α,φ,η,γ,D), we denote the posterior probability density of the parameters as:

$$P\left(w,\alpha ,\varphi ,\eta ,\gamma |\hat{\Omega }\right)\propto P(\hat{\Omega }|\Omega \left(w,\alpha ,\varphi ,\eta ,\gamma ,D\right))\times P(w)P(\alpha )P(\varphi )P(\eta )P(\gamma ),$$ (6)

where P(w), P(α), P(φ), P(η), and P(γ), are prior distributions. All parameters are given (half-)Gaussian priors except for $\alpha_{2},$ which is uniform on (0,2), and *w*, for which we use an independent Dirichlet of dimension *K* for each sample (see Table A1 for specifics). Parameters are independent between layers. We use Hamiltonian Monte Carlo as implemented in STAN (Hoffman and Gelman 2014; Carpenter 2015; Stan Development Team 2015, 2016) to estimate the posterior distribution on the parameters. Our R package, conStruct (for “*con*tinuous *struct*ure”), functions as a wrapper around this inference machinery.

### Relationship of this model to nonspatial structure models

A nice feature of our approach is that the model described in Eq. 4 contains a nonspatial assignment model as a special case (see *Models*, *Parameters*, *and Priors* for a more in-depth discussion). By setting $\alpha_{0}^{\left(k\right)}$ to zero for all *k*, we obtain a nonspatial model in which each cluster has its own allele frequency at each SNP, and individuals draw a proportion of their ancestry from each cluster. This model is very similar to that of STRUCTURE (Pritchard *et al.* 2000) and related models (*e.g.*, Alexander *et al.* 2009); the main difference is that our likelihood assumes that allele frequencies are normally distributed around their expectations, while the standard assignment methods assume that the error is binomially distributed (Engelhardt and Stephens 2010). (We make this approximation for the substantial advantages in computational speed.) The second difference is that, in the original STRUCTURE model, allele frequencies at each locus are independently drawn for each cluster (Pritchard *et al.* 2000), while in conStruct’s nonspatial model, it is more natural to envision each cluster’s allele frequency as being drifted away from a single, global allele frequency. This makes our model more closely related to the “*F*-model” prior for allele frequencies of Falush *et al.* (2003). These differences in the underlying model could in principle result in different behavior, but below we show that the nonspatial model indeed produces similar results to ADMIXTURE, and use this fact to compare the fit of the different models—spatial *vs.* nonspatial, across different values of *K*—by comparing their performance in a common framework.

### Choice of layer number and cross-validation

There are a number of reasons why there is no true (or right) number of layers for real datasets, discussed further in the *Discussion*. However, it is still important to assess whether additional layers (larger *K*) meaningfully model patterns in the data or merely explain spurious variation introduced by noise—in other words, whether additional model complexity provides significant explanatory power. Toward that end, we have implemented a method for statistically comparing conStruct results across different values of *K* and between the spatial and nonspatial models.

Several approaches have been used as model choice criteria for the number of discrete clusters in population genetic data, including: comparisons of the likelihood of the data across different values of *K*, with various criteria on how to choose a single value (*e.g.*, Evanno *et al.* 2005), or with information theoretic penalizations such as Akaike information criterion (AIC) or Bayesian information criterion (BIC; *e.g.*, Alexander *et al.* 2009); comparisons of the marginal likelihood, generated either via various approximations (*e.g.*, Pritchard *et al.* 2000) or via thermodynamic integration (Verity and Nichols 2016); or inference using a Dirichlet process prior (Huelsenbeck and Andolfatto 2007). See Verity and Nichols (2016) for a discussion of these approaches and comparison between several methods.

We use cross-validation [similar in spirit to Alexander and Lange (2011)] to attack this problem. To do this, we use a “training” partition of the data (in practice, a random 90% subset of the loci) to estimate the posterior distribution of the parameters, and then calculate the log-likelihood of the remaining “testing” loci, averaged over the posterior. Prediction accuracy of a particular value of *K* is then measured using this log-likelihood, averaged over a number of independent data partitions. The best model is judged to be the simplest one with significantly better predictive accuracy than others (see *Cross validation procedure* for more on our cross-validation procedure). In general, larger values of *K* allow the model more flexibility, and thus increases the likelihood of the training partition, but this improvement in the likelihood will plateau (or even peak), as above a certain *K* the model only fits noise specific to the training data rather than generalizable patterns. At any value of *K*, support for the spatial model over the nonspatial model means that isolation by distance is likely a feature of the data.

Cross-validation provides a valuable summary of how much explanatory power is added by spatial structure within each layer, and each additional layer. However, we remind users that “statistical significance does not imply real-world significance,” and so small but statistically significant differences between models should not be relied on too strongly.

Another way to describe the practical significance of additional layers is to calculate each layer’s relative contribution to total covariance, and to choose a value of *K* where all layers have a contribution above some cutoff (*e.g.*, 0.1%). The Dirichlet prior on admixture proportions is quite harsh against intermediate admixture values (see Table A1), encouraging the model to “not use” unnecessary layers if they are present in the model, so that they will have a low contribution to overall covariance.

To calculate layer contributions, we use the following alternative description of our covariance model: the genomes of any pair of individuals *agree* with some background probability at a locus, but this probability of agreement is increased on any segment of genome that both have inherited from the same layer (the amount it increases depends on how far apart they are geographically and on the decay of isolation by distance). We use this characterization to quantify the relative contributions of each layer by computing the average contribution to increased probability of agreement as described in *Calculating layer contributions*. This layer contribution is similar to the “ancestry contribution” proposed by Raj *et al.* (2014). However, each of our layers can induce a different amount of covariance between samples embedded in them, so we take that into account when calculating each layer’s contribution to the whole.

### Data availability

The method conStruct is implemented as an R package, and is available for installation at https://github.com/gbradburd/conStruct. Scripts for generating and analyzing all simulated and empirical datasets, as well as the datasets themselves, are also available at the same site, and additionally have been archived at Data Dryad (doi: 10.5061/dryad.5qj7h09). Supplemental material available at Figshare: https://doi.org/10.25386/genetics.6840629.

## Results

### Simulations

**Figure A1:**
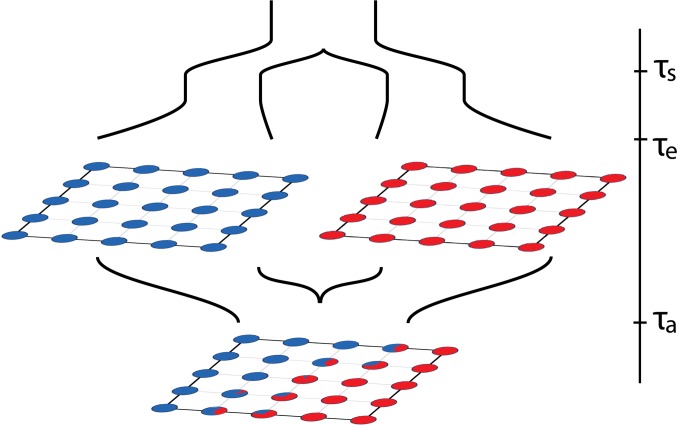
Schematic of how we simulate datasets with continuous and discrete differentiation, using K=2 as an example. Going forward in time, the *K* populations split from a common ancestor at time $\tau_{\text{s}},$ then expand to each colonize a lattice of demes with nearest-neighbor symmetric migration at time $\tau_{\text{e}},$ then finally at time $\tau_{\text{a}}$ collapse into a single lattice consisting of demes with ancestry entirely in one or the other of the populations, or admixed between them.

To test the method, we first generated data using the coalescent simulator ms (Hudson 2002). In each simulation, we split a single ancestral population into *K* subpopulations $\tau_{\text{s}}$ units of coalescent time in the past, and at time $\tau_{\text{e}}$ in the past, each of these discrete populations instantaneously colonized a separate 6×6 square lattice of demes. Migration on each lattice was to nearest neighbors (eight neighbors, including diagonals). Finally, at time $\tau_{\text{a}}$ in the past, we collapsed those *K* discrete layers into a single grid of demes, choosing various amounts of admixture from these different layers (see Figure A1), with randomly distributed but spatially autocorrelated admixture proportions. See *Simulation details* for more details, including parameter values used. We simulated datasets using K=1, 2, and 3 layers; in each simulation we sampled 10,000 unlinked loci from each of 20 haploid individuals from every deme. We then ran both spatial and nonspatial conStruct analyses on each simulated dataset with *K* between 1 and 7, and compared predictive performance of the models using cross-validation with 10 replicates. For comparison, we also analyzed each simulated dataset using ADMIXTURE (Alexander *et al.* 2009) with *K* between 1 and 7, and compared models using ADMIXTURE’s cross-validation procedure with 50 folds.

**Figure 2:**
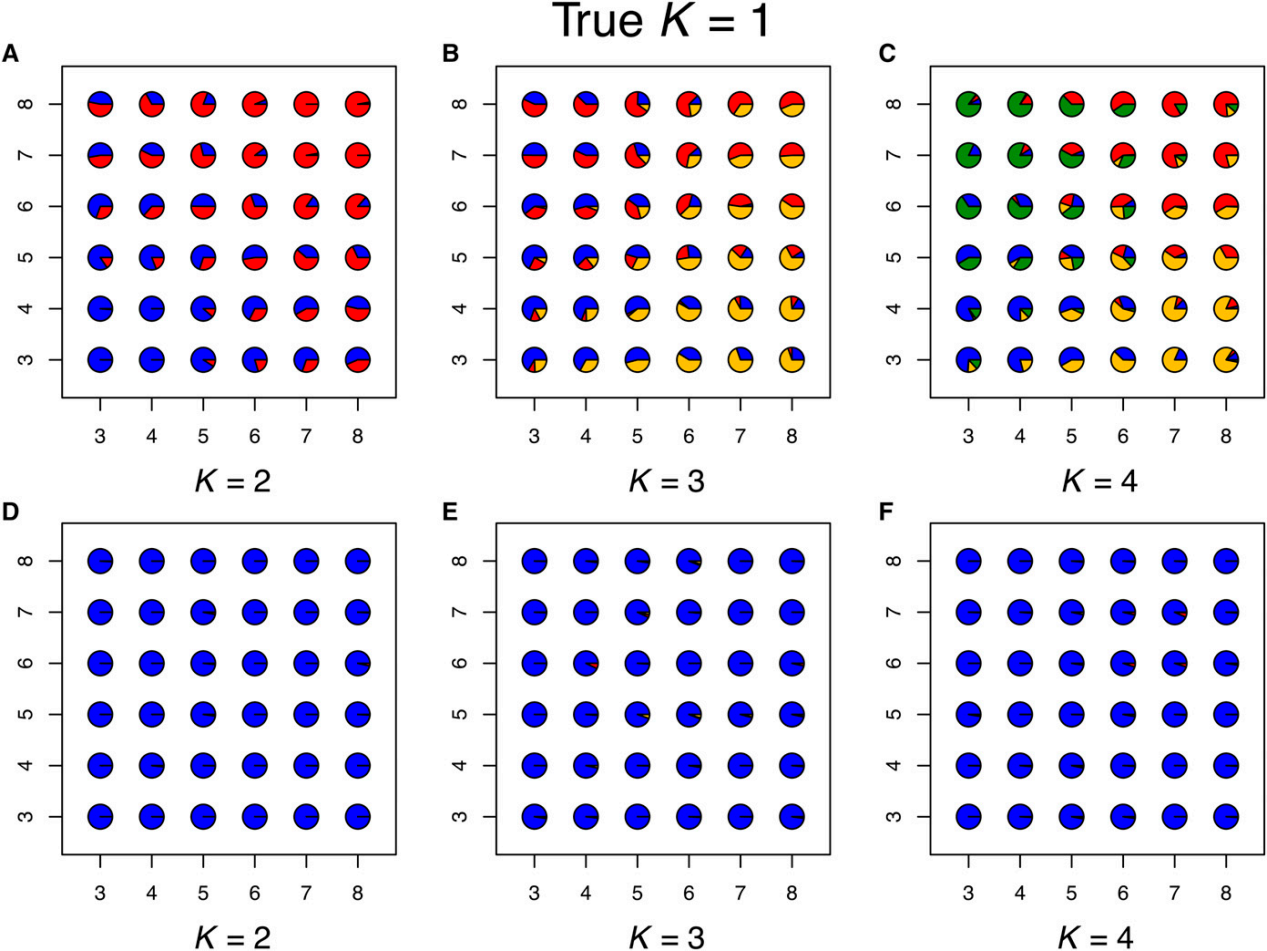
Results for data simulated using K=1, showing maps of admixture proportions estimated using the nonspatial conStruct model for K=2 through 4 [(a)–(c); top row] and the spatial conStruct model for K=2 through 4 [(d)–(f); bottom row]. As there is only a single layer in the simulation, no populations should be admixed, which is accurately depicted by the spatial model (second row), while the nonspatial model creates spurious clusters (first row).

With these simulations, spatial conStruct does not create spurious discrete groupings when there are none: Figure 2, Supplemental Material, Figure S1, Figure S2, and Figure S3 show that subsequent layers beyond the number used for simulation are unused. When data simulated with K=1 are analyzed with K>1, the additional layers contribute very little to any population. Even when the spatial model is run with K=7, the inferred admixture proportions are nearly identical to those estimated under the true value of *K* for each simulation. Moreover, the method infers the true admixture proportions with high accuracy, tight precision, and good coverage (Figure S4 and Figure S5).

In contrast, the nonspatial model describes geographic variation using gradients of admixture between increasingly many discrete clusters to better approximate the continuous, spatial patterns of relatedness (Figure 2, Figure S6, Figure S7, and Figure S8). The ADMIXTURE results are qualitatively similar, as shown in Figure S9, Figure S10, and Figure S11. Each nonspatial cluster is genetically more similar within itself than it is to other clusters, but we know that these boundaries are arbitrary, because the data were simulated without them.

**Figure 3:**
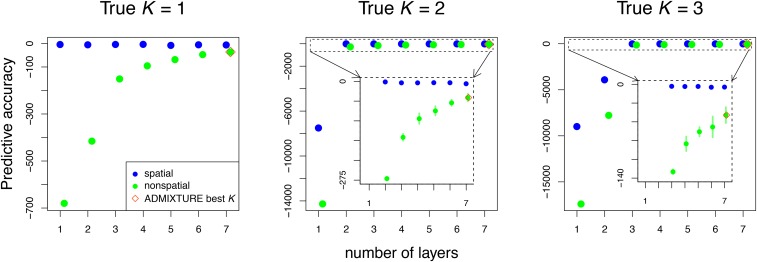
Cross-validation results for data simulated under K=1, K=2, and K=3, comparing the spatial and nonspatial conStruct models (in blue and green, respectively) run with K=1 through 7, with 10 cross-validation replicates. The inset plots zoom in on cross-validation results outlined in the dotted boxes. The spatial model shows better model fit at every value of *K*. The vermillion diamond indicates the value of *K* selected on the basis of lowest cross-validation error among ADMIXTURE models. In all simulations, the preferred ADMIXTURE model was that with the largest number of clusters.

The spatial model’s better fit is reflected by increased predictive accuracy: as shown in Figure 3, across all models and choices of *K*, the spatial model is correctly preferred over the nonspatial model. As desired, predictive accuracy of the spatial model increases until the true value of *K*, and then plateaus or declines (Figure 3, Figure S12, Figure S13, and Figure S14). Predictive accuracy of the nonspatial model increases as subsequent clusters are added up to K=7 (the largest number tested), although gains are greatest as layers below the true number are added. The same holds true for the ADMIXTURE cross-validation results, in which models that have the largest number of clusters are preferred over all other models, as shown in Figure 3 (vermilion diamonds), and, in more detail, in Figure S15.

**Figure 4:**
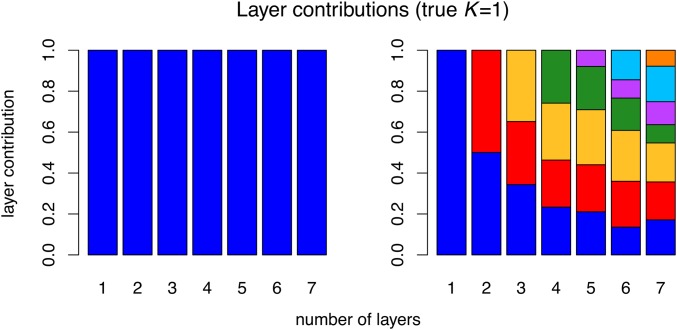
Results for data simulated using K=1, showing layer/cluster contributions (*i.e.*, how much each layer/cluster contributes to total covariance), from conStruct runs using K=1 through 7 for the spatial model (left), and the nonspatial model (right). In each run of the spatial model, a single layer explained nearly all the covariance (additional bars are present but not visible).

The unimportance of spurious layers can be seen in plots of layer contributions (Figure 4, Figure S16, and Figure S17). In the spatial analyses, once we pass the true *K*, subsequent layers add little in terms of (co)variance explained; in contrast, additional clusters in the nonspatial analyses continue to contribute substantially.

### Empirical applications

To further demonstrate the utility of this method, we also applied conStruct to empirical population genomic data from two systems: a contact zone between two poplar species in northwestern North America, and a large North American sample of black bears.

### Poplars

#### Study system and questions:

Trees in the genus *Populus* (poplars, aspens, and cottonwoods) are distributed throughout the Northern Hemisphere; species in the genus regularly co-occur and, where they do, they frequently hybridize (Eckenwalder 1984; Cronk 2005).

*Populus trichocarpa*, the black cottonwood, and *Populus balsamifera*, the balsam poplar, have a broad zone of overlap in the Pacific Northwest, where they are hypothesized to hybridize (Geraldes *et al.* 2014; Suarez-Gonzalez *et al.* 2016). Both species are sampled over a large geographic region, and show spatial patterns of genetic and phenotypic variation (Slavov *et al.* 2012; McKown *et al.* 2014), making the system well-suited for application of our method. We organize the results of our analyses around the following questions:

1. To what degree has hybridization blurred the boundaries between *trichocarpa* and *balsamifera*? (As an extreme case, does genetic differentiation support these as separate species, as opposed to a single cline of ancestry?)
2. Does the only significant boundary of population structure fall along the species boundary (if any), or is there substructuring within species?
3. Does the strength of isolation by distance differ between inferred layers? This may indicate, *e.g.*, different speeds of postglacial expansion or primary modes of dispersal.

#### Data and analyses:

**Figure 5:**
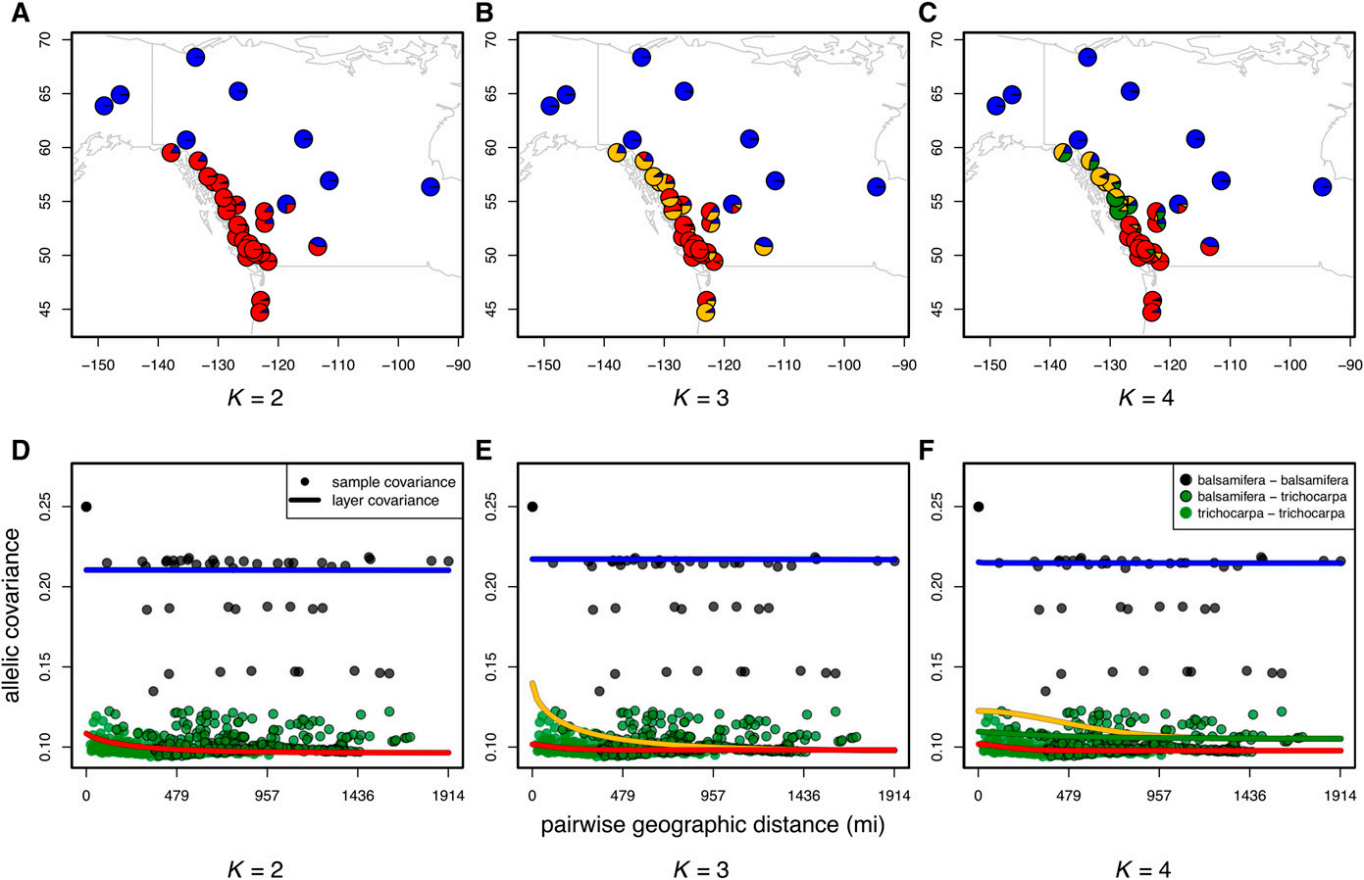
Maps of admixture proportions estimated for the *Populus* dataset using the spatial conStruct model for K=2 through 4 (a–c), as well as the corresponding layer-specific covariance curves estimated under each model (d–f).

**Figure 6:**
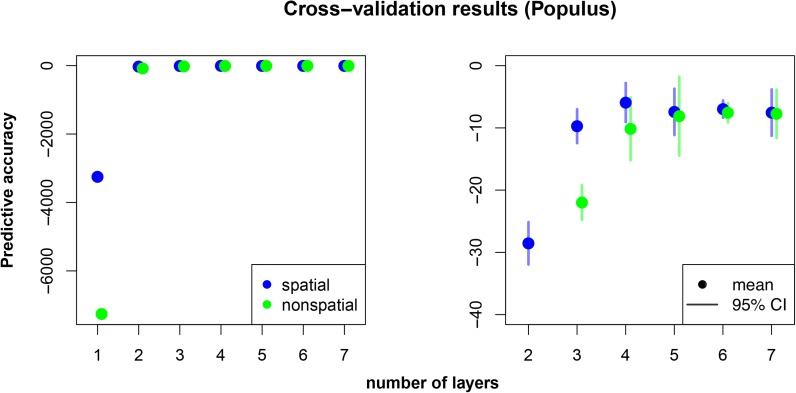
Cross-validation results for *Populus* dataset comparing the spatial and nonspatial conStruct models run with K=1 through 7 with 10 cross-validation replicates. The first panel in each row shows all results; the second panel zooms in on the results from analyses run with K=2 through 7.

We use data from Geraldes *et al.* (2014), consisting of 434 individuals sampled from 35 drainages genotyped at just over 33,000 loci (map of the sampling shown in Figure S18). The number of individuals per drainage ranged between 1 and 50, with most sampling concentrated on *trichocarpa* drainages. The data were generated using an Infinium 34K array designed for *trichocarpa* (Geraldes *et al.* 2013), and showed a strong pattern of bias in allelic dropout (the majority of missing data were from drainages with only *Populus balsamifera* individuals). To ameliorate some of the problems that arise when there is a strong bias in which data are missing, we dropped loci for which any data were missing, resulting in just over 20,200 loci retained for analysis. We then analyzed these data, grouped by drainage, using both the spatial and nonspatial conStruct models with K=1 through 7, and compared these models using cross-validation with 10 replicates. The results of all these analyses are shown in Figure 5 and Figure 6, as well as Figure S19, Figure S20, Figure S21, Figure S22, and Figure S23 in the “Supplemental Materials”. For comparison, we also ran ADMIXTURE (Alexander *et al.* 2009) with K=1 through 7, using 50-fold cross-validation to compare model performance (Figure S24 and Figure S25).

#### Results from construct:

All models with K>1 assigned the majority of each of the two species to distinct layers, with some populations drawing ancestry from multiple layers. Based on cross-validation results, we view the K=3 spatial model as a sufficient description of the data, with additional structure of uncertain significance. This provides strong support for discrete population structure between the two species, with some admixture, rather than a single, continuous cline of ancestry. At all values of K>1, discrete population structure was mostly partitioned along species lines; at values of *K* above 2, further discrete substructure was inferred within the *P. trichocarpa* samples, with no substructure within *balsamifera*. There was also strong support for isolation by distance in the dataset, but most of this signal seems to derive from the *P. trichocarpa* samples: as seen in Figure 5 d–f and Figure S21, there is almost no isolation by distance within the *balsamifera* layer ($\alpha_{D}\approx 0$). Both points are in agreement with previous work (Keller *et al.* 2010), which found low diversity within the region’s *balsamifera*, probably as the result of a recent postglacial expansion.

A consistent split between layers within *trichocarpa* fell along the “no-cottonwood belt,” a region along the central coast of British Columbia in which black cottonwood is absent (the break between yellow and red, for K≥3). The no-cottonwood belt is hypothesized to divide the species’ distribution into northern and southern groups, which, in a provenance test, were experimentally shown to display differences in ecologically relevant phenotypes (*e.g.*, pathogen resistance, Xie *et al.* 2009, 2012). At higher values of *K*, drainages at the southern tip of *trichocarpa* sampling begin to split out into their own layers, perhaps due to introgression from the southern neighbors *Populus angustifolia* or *fremontii* (Zhou and Holliday 2012; Geraldes *et al.* 2014).

#### Comparison to ADMIXTURE:

Both nonspatial conStruct and ADMIXTURE displayed the successive partitioning of space and the clines of admixture seen in the simulation results. The details of each were somewhat different (Figure S20 *vs.* Figure S24), and also differed across the replicate analyses. These differences between runs and methods may be due to noise in the different inference algorithms employed, multi-modality in the likelihood surfaces, or to model details (*e.g.*, the priors used in nonspatial conStruct, or the fact that ADMIXTURE is modeling each allele’s frequency in each cluster, rather than the covariance across all alleles). However, overall, the behavior of both methods was quite similar: each recovered the *trichocarpa*/*balsamifera* split with the first two clusters modeled, then, with higher values of *K*, used subsequent clusters to subdivide the *trichocarpa* samples into geographically restricted foci of cluster membership. Both nonspatial conStruct and ADMIXTURE strongly favored the most cluster-rich model (Figure 6 and Figure S25). In contrast, the spatial conStruct model clearly did not favor the model with the highest value of *K*, and appears to describe patterns of isolation by distance across the *trichocarpa* range quite well.

### Black bears

#### Study system and questions:

The American black bear, *Ursus americanus*, is endemic to North America and has a broad distribution across the continent. During the last glacial maximum, black bears were confined to isolated glacial refugia, from which they subsequently expanded to occupy their current range (Byun *et al.* 1997; Wooding and Ward 1997; Stone and Cook 2000; Puckett *et al.* 2015), likely leading to both continuous and discrete patterns of genetic structure. We organize our results around the following questions:

1. How many distinct populations are reflected in modern patterns of genetic variation?
2. How strong is isolation by distance within each inferred group?

Distinct populations likely represent different glacial refugia, and differing strengths of isolation by distance might indicate different levels of habitat connectivity, dispersal behavior, or different postglacial histories.

#### Data and analyses:

**Figure 7:**
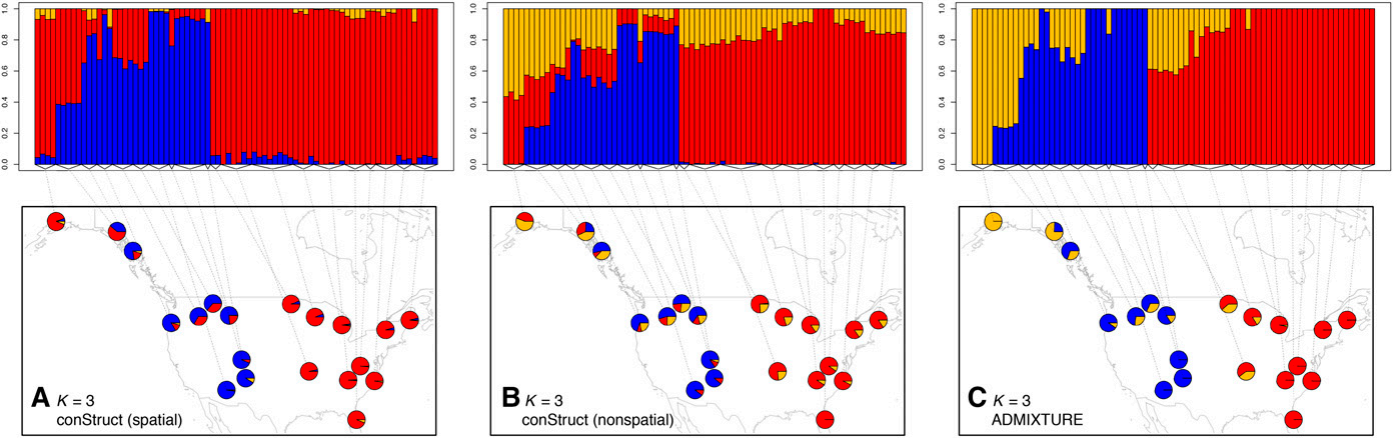
Maps of admixture proportions estimated for the black bear dataset using the spatial conStruct model (a), the nonspatial conStruct model (b), and ADMIXTURE (c) for K=3. Pies show mean admixture results across individuals within their diameter, and the admixture results for all individuals included within each group are shown in the plot above.

**Figure 8:**
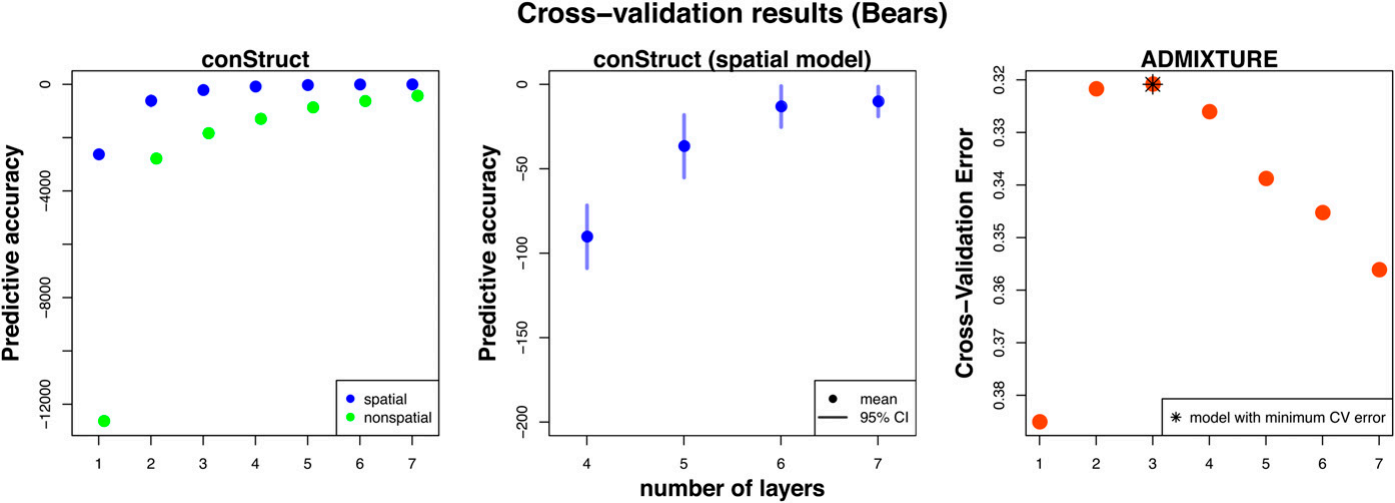
Cross-validation results for the black bear dataset, comparing spatial and nonspatial conStruct models, as well as ADMIXTURE, all run with K=1 through 7, with 10 cross-validation replicates for the conStruct analyses and 50 data-fold subsets for the ADMIXTURE analyses. The first panel in each row shows results from spatial and nonspatial conStruct models; the second panel zooms in on the results from the spatial analyses run with K=4 through 7, and the third panel shows the results for ADMIXTURE. Note that the admixture plot shows cross-validation error (rather than predictive accuracy), and that the *y*-axis has therefore been flipped for ease of comparison to the conStruct results.

**Figure 9:**
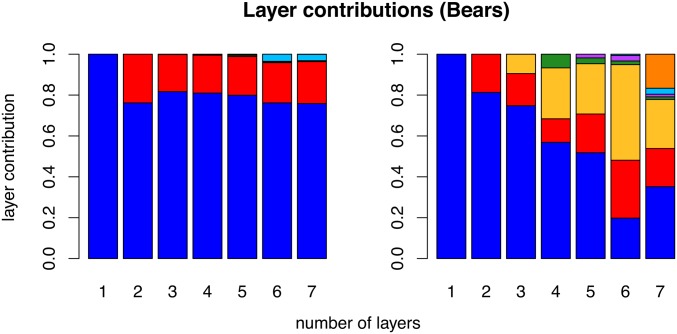
Layer/cluster contributions (*i.e.*, how much total covariance is contributed by each layer/cluster), for all layers estimated in runs using K=1 through 7 for the spatial model (left), and for all clusters using the nonspatial conStruct model (right). For each value of *K* along the *x*-axis, there are an equal number of contributions plotted. Colors are consistent with Figure 7.

We use data from Puckett *et al.* (2015), consisting of 95 individuals sampled across the United States and on the West coast of Canada, genotyped at just under 22,000 biallelic loci. The distribution of missing data across these individuals was uneven, with a few individuals representing most of the missing data, so we removed individuals with >4% missing data, resulting in a final dataset of 78 individuals. We then analyzed these data, treating individuals as the unit of analysis, using both the spatial and nonspatial conStruct models with a *K* of between 1 and 7, and compared these models using cross-validation with 10 replicates. We also ran ADMIXTURE (Alexander *et al.* 2009) on the same dataset, using K=1 through 7, and comparing models using ADMIXTURE’s cross-validation procedure with 50 data fold subsets. The results of these analyses are shown in Figure 7, Figure 8, and Figure 9, as well as in Figure S26, Figure S27, Figure S28, Figure S29, Figure S30, and Figure S31 in the “Supplemental Materials”.

#### Results from conStruct:

The results partition the sampled bears into two main groups (shown in Figure 7a): one (red) to the east of the Rocky Mountains, which also occurs in Alaska, the other primarily west of the Rockies (blue). The disjointed range of the red layer likely reflects the fact that Canada was not sampled, and so the red layer may extend through the intervening (unsampled) northern Great Plains and Canadian Shield, with the blue layer presumably then stretching up into British Columbia.

The spatial models have strong statistical support up until around K=5 or 6 (Figure 8), but additional spatial layers beyond K=2 contribute little to total covariance (Figure 9). The locations of admixed individuals are consistent with a scenario of postglacial expansion from two refugia, one in the American Southwest and one in the American Southeast, meeting near the Northwest coast of North America and the Cascade Range. However, lack of any samples from Canada and Mexico, and lack of denser sampling across northern North America, make more detailed interpretations untrustworthy. The spatial covariance functions estimated in layers beyond the first two take very large values over small spatial lags, but decay sharply after that. This feature, combined with the overall amounts and spatial patterns of ancestry in those layers, suggests that these layers are describing processes that shape genetic variation at local scales, such as inbreeding, which affects covariance between individuals within each location, but has limited impact on covariance between locations.

#### Comparison to ADMIXTURE:

Results from the nonspatial model and from the ADMIXTURE analyses clearly exhibit the tendency of nonspatial clustering algorithms to describe continuous spatial patterns of divergence using gradients of admixture between clusters. For example, in Figure 7b, the third cluster (in gold) exhibits a clear East-West gradient that overlays the discrete structure between the Southwest cluster and the Southeast. The results from ADMIXTURE are not identical to those obtained using the nonspatial conStruct model, but they do show the same tendency: *e.g.*, at K=3 — the preferred model from the cross-validation analysis shown in Figure S31 — ADMIXTURE splits the westernmost Alaskan samples out of the cluster with the eastern samples, and at K=4, it subdivides the eastern cluster into two geographically partitioned groups (Figure S30). Interestingly, for the nonspatial model implemented in ADMIXTURE, the preferred model has a smaller *K* (K=3) than that of the spatial models with best cross-validation performance in conStruct (K=5 or 6). This discrepancy likely stems from the different features introduced in layers beyond K=2 in the two models: conStruct uses small contributions of new layers to model very local drift, while ADMIXTURE moves to geographically finer subdivisions.

Even at K=3, ADMIXTURE invokes clusters to describe what seems to be a continuous spatial pattern of genetic variation, which conStruct describes using only two spatial layers. The third cluster in the ADMIXTURE analysis at K=3 (shown in gold in Figure S30b), shows strong spatial autocorrelation in admixture proportions, as would be expected if it is describing continuous spatial differentiation. The allelic covariances plotted against distance (see Figure S32) provide more information on ADMIXTURE’s lack of fit: covariance between Eastern bears falls off gradually rather than abruptly with distance, indicating a residual pattern best explained by isolation by distance within layers. In addition, the covariance between bears assigned to ADMIXTURE’s gold and red layers (the furthest Northwestern and Eastern bears, respectively) appears to be a natural extension of the decay of covariance with distance, falling to only slightly lower values than covariances between other widely separated pairs of Eastern sampling locations.

Across all values of *K* for which we ran conStruct, we see strong support for the spatial model over the nonspatial model (Figure 8). This pattern may resolve a discrepancy between our results and previous analyses that split Alaskan and British Columbian bears out into their own cluster with an inferred Beringian glacial refugium (Byun *et al.* 1997; Stone and Cook 2000; Puckett *et al.* 2015). Our model, which explicitly incorporates a spatial decay of relatedness, allows somewhat genetically differentiated individuals that are sampled far from one another to belong to the same layer, instead of splitting these individuals out into successive clusters (*e.g.*, Figure S26d *vs.* Figure S27d).

## Discussion

In this paper, we have presented a statistical framework, conStruct, for simultaneously modeling continuous and discrete patterns of population structure. By employing the sensible default assumption that relatedness ought to decay with geographic distance, even within a population, we avoid erroneously ascribing population differentiation to discrete population clusters. To aid comparison between models, we present a cross-validation approach as well as a way to describe the contribution of each spatial layer to the model (but caution against overly strict interpretation of either).

The method performs well on simulated data: we accurately infer the admixture proportions used to simulate the data and accurately pick the simulating model as the best model using our cross-validation procedure. Two empirical applications of conStruct to samples of North American poplars and black bears yield reasonable results, and demonstrate that, by acknowledging isolation by distance, real datasets can be better described using fewer layers.

The proposed method combines the utility of model-based clustering algorithms with a model of isolation by distance. We anticipate that conStruct will be useful for identifying populations and determining ancestral origins of sampled individuals, especially when the populations exhibit geographic patterns of relatedness.

### Comparison to nonspatial model-based clustering

Above, we showed that (a) the nonspatial conStruct model recapitulates results of other, commonly used nonspatial clustering methods, and (b) conStruct can concisely capture spatial structure, which is common within populations. Given this, when should methods without spatial capability be used? One advantage these have over conStruct is speed when the number of samples is large. Although conStruct’s computation time is independent of the number of loci included in the dataset (after the initial calculation of the allelic covariance), it currently scales poorly with number of samples. The computationally limiting step is the inversion of the covariance matrix, which scales more than quadratically with the number of samples, whereas computation time for, *e.g.*, STRUCTURE, scales linearly with number of samples.

For a relatively small number of samples, conStruct can be much faster than existing nonspatial Bayesian clustering methods. On a desktop machine, using a single 4.2 GHz Intel Core i7 processor, an analysis of the black bear dataset (78 samples, 21,000 loci) running conStruct’s spatial model with four layers for 5000 Markov chain Monte Carlo (MCMC) iterations (which was more than sufficient for convergence) took 2.8 hr. For almost any size dataset, the maximum likelihood algorithm implemented in ADMIXTURE is quite a bit faster than conStruct: running ADMIXTURE on the bear dataset over all values of *K* from 1 to 7, including 50-fold cross-validation for each value of *K*, took only 6.6 min. It should also be noted that there may be situations when the binomial-based model underlying ADMXITURE performs better than our Gaussian-based model, *e.g.*, when clusters differ at only a few strongly differentiated loci, although we have not investigated this possibility.

### Choosing the “best” number of layers

Although we recognize the utility of choosing a single, “best” value of *K*, and using only that analysis to communicate results, we emphasize that the choice of best *K* is always relative to the data in hand and the questions to be answered. From a statistical perspective, unless the data were generated under the model itself, the support for larger values of *K* is likely to increase with increasing amounts of data. In the limit of infinite data, the best value of *K* may be the number of samples included in the dataset (Patterson *et al.* 2006).

From a biological perspective, it is important to stress that patterns of relatedness between individuals and populations are shaped by complex spatial and hierarchical processes. All individuals within a species are related to one another in some way, and summarizing those patterns of relatedness with a single value of *K* may be reductive or misleading. We therefore encourage users to perform analyses across different values of *K* and observe which layers split out at what levels (this is conceptually similar to taking successively shallower cross-sections of the population phylogeny), and also to take the results of the proposed cross-validation procedure with a large grain of salt. Calculating layer contributions may also be a useful heuristic, as it can reveal layers with statistical support but small biological import.

Although we believe our model adds spatial realism to the groups used by clustering methods, it is important to note that the layers detected by our method do not necessarily correspond to distinct, ancestral populations; nor does a nonzero admixture proportion indicate that admixture (*i.e.*, gene flow) must have occurred. Both groupings and admixture proportions should be viewed as hypotheses that should be subject to further testing (for an indepth discussion of these points, see Falush *et al.* 2016).

### Implications for management and conservation

Because isolation by distance is common, a likely result of applying conStruct to existing data is that populations previously identified as distinct using nonspatial clustering methods may be grouped into the same layer. This “lumping” might better reflect the demographic history of these populations, but may not contradict the genetic distinctness implied by the nonspatial clustering. This genetic distinctness—rather than shared history—may be more relevant for management decisions and conservation policy, which are often predicated on the identification of discrete “management units” identified using genetic data (Moritz 1994; Waples 1998; Moritz *et al.* 2002).

It is therefore important to stress that individuals sampled from the same conStruct layer may be quite genetically diverged from one another, perhaps especially at loci underlying adaptive traits, and that a conStruct layer may still contain multiple distinct management units worthy of independent protections. For instance, although both the Alaskan and Eastern Black Bears draw most of their ancestry from the same conStruct layer, they are separated by a great distance, and may therefore differ substantially from each other (although less than from the Western bears, as measured by average covariance). Alternatively, the inclusion of multiple management units into a single conStruct layer may occur if these populations are currently (or were recently) exchanging migrants, and thus might emphasize the importance of maintaining habitat corridors, or of implementing an integrated conservation plan across a geographic region.

### Allelic or genetic covariance?

The choice of allelic covariance, rather than genetic covariance, was motivated by the fact that it is less affected by sample configuration—the genetic covariance is calculated after subtracting the mean from the entire sample, which is more strongly affected by densely sampled locations. Genetic covariance is also often computed after first dividing each frequency by $\sqrt{p\left(1-p\right)},$ where *p* is the global allele frequency, with the aim of equalizing variances across loci. Our definition does not do this, and so is less affected by low-frequency alleles. Both of these changes led to better performance on test data. However, note that allelic covariance is more affected by singleton sites than the standard genetic covariance, so it may be advisable to filter these prior to analysis if they are likely to contain a large percentage of errors (Linck and Battey 2017).

### Caveats and considerations

There are a few important caveats to consider in the interpretation of conStruct results. First, we have modeled allelic covariance within a layer as a spatial process. Although there is flexibility built into the model about the shape of that covariance, inference may be misleading if the sampling geography departs radically from the way the sampled organisms disperse (or have dispersed) on their landscape. For example, if we were to run a conStruct analysis using geographic distances between sampled individuals of greenish warblers (Irwin *et al.* 2001) or *Ensatina* salamanders (Wake and Schneider 1998)—two canonical examples of ring species—we might get misleading results. This is because distance between locations on either side of the species’ distributions (across the Tibetan plateau and the Central Valley, respectively) is not representative of the path traversed in the coalescence of a pair of alleles sampled at those locations.

A second caveat is that, in some instances, membership in the same layer may not mean that samples are particularly related. If covariance within a layer decays sharply with distance, and the layer-specific relatedness parameter $\varphi^{\left(k\right)}$ is low, individuals separated by a large spatial distance may be in the same layer but have very low pairwise relatedness. It is possible that this is happening in Figure S19. At K=3, the southernmost populations of *P. trichocarpa* are in the gold layer, whose other neighbors are to the north, with an intervening group of populations in the red layer, and at K=5, those southernmost samples split out and become their own layer. Furthermore, note that in this case $\alpha_{0}^{\left(k\right)}$ and $\varphi^{\left(k\right)}$ are confounded, so differences in *φ* between layers should not be overinterpreted. Again, we encourage users to run analyses across multiple values of *K* and to examine the spatial covariance functions within layers when interpreting results.

### Extensions and future directions

There are several ways in which the model described in this paper might be extended or improved. For example, we currently assume that all layers within a model are equally unrelated (a star population phylogeny, although the branches can have different lengths thanks to the $\varphi^{\left(k\right)}$ parameter), similar to the F-model of Falush *et al.* (2003). However, we could extend the existing model by implementing a relatedness structure between the layers by, for example, estimating a population phylogeny between them (*e.g.*, Pickrell and Pritchard 2012).

In addition, here we have assumed that samples have known geographic coordinates, and that they draw ancestry from layers only at those sampled locations. A natural extension would be to attempt to “geo-locate” the ancestry of samples without geographic coordinates (Wasser *et al.* 2004). We could also imagine letting samples draw ancestry from other geographic coordinates, as we have done in a previous approach (Bradburd *et al.* 2016) to model long distance dispersal. We could even allow entire layers to bud off of a particular location on another layer. This would enable more explicit modeling of range expansion or domestication, in which a set of individuals are thought to have ancestry that originated from a particular geographic location embedded in a larger pattern of isolation by distance.

A final direction would be to model relatedness within a layer as a spatiotemporal process, in which covariance decays both with distance in space and in time. As the number of genotyped historical or ancient samples increases, it is becoming possible to ask whether there is genetic continuity at a point in space across time, or whether populations are being replaced (Lazaridis *et al.* 2014; Haak *et al.* 2015; Slatkin and Racimo 2016; Nielsen *et al.* 2017; Schraiber 2017; Joseph and Pe’er 2018). However, we expect allele frequencies to change through time in a population, even without replacement, simply due to drift. Therefore, a natural way to test for population replacement is to estimate the rates at which relatedness within a layer decays with time in the same way we do in the current model with space, in which case a change in discrete population structure across space is comparable to population replacement across time.

## Appendices

### Model Rationale: Drift, Admixture, and Space

Here we sketch a simple model of allele frequencies and their covariances, to justify the form given in the main text.

#### Drift

We first provide a simple model of allele frequencies within a layer. Imagine a sample *i* that draws all of its ancestry from layer *k*. The allele frequency in sample *i* at locus ℓ, denoted $F_{i,\ell },$ can be written as the sum

$$F_{i,\ell }=\epsilon_{\ell }+\Delta_{\ell }^{\left(k\right)}+\Delta_{\ell }^{\left(k,i\right)}+\Delta_{\ell }^{\left(i\right)}.$$ (7)

The first term is the ancestral allele frequency $\epsilon_{\ell }$ shared by all samples; the second is the deviation from that ancestral frequency due to drift in the ancestral population of the *k*th layer, which is shared by all samples within the layer. The third term is the deviation of the *i*th sample away from the *k*th layer mean due to the spatial process of drift and migration within the layer. The final term is the deviation specific to the *i*th sample, which captures drift not shared by all samples at the population level (*i.e.*, subpopulation-specific drift due to, *e.g.*, inbreeding). We will assume that these four deviations are all uncorrelated with each other.

If we have two samples *i* and *j* drawn from layer *k*, their covariance across loci will be

$$\text{Var}\left(\epsilon \right)+\mathrm{Var}\left(\Delta^{\left(k\right)}\right)+\mathrm{Cov}\left(\Delta^{\left(k,i\right)},\Delta^{\left(k,j\right)}\right)+\delta_{i=j}\mathrm{Var}\left(\Delta^{\left(i\right)}\right),$$ (8)

where the quantity $\delta_{i=j}$ is an indicator variable that equals 1 when *i* is equal to *j* and 0 otherwise, as in Eq. 4.

#### Admixture

The model above describes the simple case in which samples draw 100% of their ancestry from only a single layer each. To accommodate admixture between layers, we model sampled genomes as drawn from allele frequencies that are weighted averages of the local frequencies in each layer from which they draw ancestry. The weights, $w_{i}^{\left(k\right)},$ describe the “admixture proportion” of sample *i* in layer *k*. These can be interpreted as the proportion of the genome in the *i*th sample that came from the *k*th layer (or the probability that an allele at a locus is drawn from layer *k*), so that $\sum_{k=1}^{K}w_{i}^{\left(k\right)}=1$ for each *i*. The allele frequency in the *i*th sample at the ℓth locus can therefore be written as:

$$F_{i,\ell }=\epsilon_{\ell }+\sum_{K}w_{i}^{\left(k\right)}\left(\Delta_{\ell }^{\left(k\right)}+\Delta_{\ell }^{\left(k,i\right)}\right)+\Delta_{\ell }^{\left(i\right)},$$ (9)

and so the covariance between *i* and *j* across loci is

$$\Omega_{i,j}=\mathrm{Var}\left(\epsilon \right)+\sum_{k=1}^{K}w_{i}^{\left(k\right)}w_{j}^{\left(k\right)}\left(\text{Var}\left(\Delta^{\left(k\right)}\right)+\text{Cov}\left(\Delta^{\left(k,i\right)},\Delta^{\left(k,j\right)}\right)\right)+\delta_{i=j}\mathrm{Var}\left(\Delta_{i}\right).$$ (10)

#### Space

Under our nonspatial model, we assume that $\mathrm{Cov}\left(\Delta_{\ell }^{\left(k,i\right)},\Delta_{\ell }^{\left(k,j\right)}\right)=0,$ so that the only additional covariance between *i* and *j* (above that induced by a shared ancestral frequency at each locus) is due to the drift in the ancestral population of their layer (the variance of which is $\varphi^{\left(k\right)}$). Under our spatial model we assume that some of the covariance in allele frequencies between *i* and *j* decays as a function of the geographic distance between the pair, $D_{i,j},$ so that

$$\text{Cov}\left(\Delta_{\ell }^{\left(k,i\right)},\Delta_{\ell }^{\left(k,j\right)}\right)=\alpha_{0}^{\left(k\right)}\times \left(\exp\left(-{\left(\alpha_{D}^{\left(k\right)}D_{i,j}\right)}^{\alpha_{2}^{\left(k\right)}}\right)\right).$$ (11)

We note that this form is chosen for flexibility and convenience, and not because it matches any explicit population genetic model of isolation by distance.

### Allelic Covariance and Inference

Here we go into further detail about both the allelic covariance we model and the modeling framework we use.

#### Allelic covariance

To see why Eq. 1 and Eq. 2 for the allelic covariance are equivalent, pick a random locus and let *A* and *B* be randomly drawn alleles at that locus from populations *i* and *j* respectively. Suppose these are each coded as “0” or “1” (where “0” denotes a reference allele), but we randomly “flip” this coding, so that we let X=A and Y=B with probability 1/2, but otherwise we let X=1−A and Y=1−B. These are $X_{i}$ and $X_{j}$ in Eq. 1, so that ${\hat{\Omega }}_{i,j}=\mathrm{cov}\left[X,Y\right].$ The random allele flipping makes the value of $\hat{\Omega }$ independent of the choice of reference allele. By conditioning on the flip, and using the fact that E[X]=E[Y]=1/2, Eq. 2 comes from the observation that

cov[X,Y]=E[(A−1/2)(B−1/2)]. (12)

Thanks to averaging over choice of alleles, the within-population allelic variance in sample *i*, ${\hat{\Omega }}_{i,i},$ is the variance of a series of Bernoulli(1/2) draws across loci, and therefore ${\hat{\Omega }}_{i,i}=1/4$ for every sample *i*. Averaging over choice of reference allele therefore removes some information about factors acting within populations that might otherwise leave signatures in the genetic covariance, such as population size, extent of inbreeding, and history of bottlenecks. However, as our model is focused on modeling *co*variances between samples as the outcome of some spatial process, we count this a minor loss.

#### Likelihood

If allele frequency deviations are well approximated by a Gaussian, their sample allelic covariance is a sufficient statistic, so that calculating the likelihood of their sample allelic covariance is the same as calculating the probability of the frequency data up to a constant. We can therefore model the covariance of the sample allele frequencies, $\hat{\Omega }\text{,}$ as a draw from a Wishart distribution with degrees of freedom equal to the number of loci *L* across which the sample allelic covariance is calculated:

$$\hat{\Omega }∼W\left(L\Omega ,L\right)$$ (13)

where W is the Wishart likelihood function.

A benefit of directly modeling the sample allelic covariance is that, after the initial calculation of the sample covariance matrix, the computation time of the likelihood is not a function of the number of loci, so inference can be done using whole genome data.

### Models, Parameters, and Priors

Here we discuss the different models implemented in this paper and give the priors we place on model parameters.

#### Spatial *vs.* nonspatial

In this paper, we discuss two types of models, spatial and nonspatial, each of which can be implemented with different numbers of layers/clusters. The spatial model is parameterized as in Equation 10, and the nonspatial model is a special case of the spatial model with all α parameters set to 0. The nonspatial model therefore has 3K fewer parameters than the spatial model, because there are three α parameters that describe the continuous differentiation effect of distance in each layer.

#### Single layer

Each of these models can be run with a single layer (K=1), in which case the layer-specific covariance parameter $\varphi^{\left(k\right)}$ and the global covariance parameter γ become redundant. The single-layer model is therefore a special case of the multi-layer model, in which we set φ to zero. For the spatial model, the single-layer parametric covariance is:

$$\Omega_{i,j}=\text{Var}\left(\epsilon \right)+\alpha_{0}^{\left(k\right)}\times \left(\exp\left(-{\left(\alpha_{D}^{\left(k\right)}D_{i,j}\right)}^{\alpha_{2}^{\left(k\right)}}\right)\right)+\delta_{i=j}\mathrm{Var}\left(\Delta_{i}\right),$$ (14)

and for the nonspatial model, it is:

$$\Omega_{i,j}=\mathrm{Var}\left(\epsilon \right)+\delta_{i=j}\mathrm{Var}\left(\Delta^{\left(i\right)}\right).$$ (15)

#### Priors

We use a Bayesian approach to parameter inference. A table of all parameters, their descriptions, and their priors is given in Table A1.

**Table A1:** List of parameters used in the conStruct model, along with their descriptions and priors

Parameter	Description	Prior
γ	Global covariance due to shared ancestral frequency	$\gamma ∼N\left(\mu =\mathrm{Var}(\bar{f}),\sigma =0.5\right)$
$\alpha_{0}^{\left(k\right)}$	Controls the sill of the covariance matrix in layer *k*	$\alpha_{0}^{\left(k\right)}∼N\left(\mu =0,\sigma =1\right)$
$\alpha_{D}^{\left(k\right)}$	Controls the rate of the decay of covariance with distance in layer *k*	$\alpha_{D}^{\left(k\right)}∼N\left(\mu =0,\sigma =1\right)$
$\alpha_{2}^{\left(k\right)}$	Controls the shape of the decay of covariance with distance in layer *k*	$\alpha_{2}^{\left(k\right)}∼U\left(0,2\right)$
$\eta_{i}$	The nugget in population *i* (population specific drift parameter)	$\eta_{i}∼N\left(\mu =0,\sigma =1\right)$
$\varphi^{\left(k\right)}$	Layer-specific shared drift in layer *k*	$\varphi^{\left(k\right)}∼N\left(\mu =0,\sigma =1\right)$
$w_{i}$	Admixture proportions sample *i* draws across *K* layers	$w_{i}∼\text{Dir}\left(\alpha_{1}\ldots \alpha_{K}=0.1\right)$

The mean of the Normal prior on γ, $\text{Var}\left(\bar{f}\right),$ is the variance of the sample mean allele frequencies across loci.

### Cross-Validation Procedure

We employ a Monte Carlo cross-validation approach for model comparison (Picard and Cook 1984). This procedure generates a mean predictive accuracy for each model and each value of *K*, as well as a confidence interval around that mean, which can then be used for model comparison or selection. Briefly, we follow the following procedure:

1. For each of *X* replicates:
  - partition the allele frequency data into a 90% “training” partition ($F_{1}^{x}$) and a 10% “testing” partition ($F_{2}^{x}$).
  - run our inference procedure using the training partition to estimate model parameters $\theta_{mk}$ for 2K models:
    i. *m*: the spatial and the nonspatial model.
    ii. *k*: the number of layers/clusters 1 through *K*.
  - calculate the mean log likelihood of the testing data partition over the posterior distribution of training-estimated parameters for each model ($\bar{ℒ}\left(F_{2}^{x}|\theta_{mk}\right),$ henceforth ${\bar{ℒ}}_{xmk}$).
  - generate standardized mean log likelihoods, $Z_{xmk},$ across all models run on this data partition:
    i. identify the highest mean log likelihood, ${\bar{ℒ}}_{x}^{\max}$ across *all* 2K models.
    ii. subtract ${\bar{ℒ}}_{x}^{\max}$ from ${\bar{ℒ}}_{xmk}$ for each model, such that the standardized log likelihood, $Z_{xmk},$ of the best model is 0, and <0 for all inferior models.
2. For each *model* (*i.e.*, each combination of *m* and *k*) calculate the mean (${\bar{Z}}_{mk}$) standardized log likelihood of the testing data partition across *X* replicates, as well as its SE ($SE_{{\bar{Z}}_{mk}}$) and 95% confidence interval (${\bar{Z}}_{mk}\pm 1.96\times SE_{{\bar{Z}}_{mk}}$).

In other words, the “predictive accuracy” shown as conStruct cross-validation results are in units of improvement in log-likelihood of that model relative to the best model for that partitioning of the data, averaged over data partitions. The standardization is necessary because different data partitions can be systematically more or less difficult to fit, resulting in greater differences in mean training data log likelihood between data partitions than between models fit to the same partition.

If the genomic coordinates of the loci are known, the training/testing partitioning should be designed to accommodate LD. Loci in strong LD are not inherited independently, so if loci from a single linkage block are included in both training and testing partitions, the independence of the test in the testing partition will be compromised because the parameters estimated from the training partition might be describing process heterogeneity or noise in a region of the genome that also has loci included in the testing partition. The best practice for cross-validation is to make sure that no loci in the testing dataset are in strong LD with, or near on the genome to, loci in the training dataset.

### Calculating Layer Contributions

Let *A* and *B* be randomly chosen alleles from samples *i* and *j* respectively, at a randomly chosen locus. Then, if we let U=2(A−1/2) and V=2(B−1/2), since *U* and *V* take the values ±1, so as in Eq. 12,

$$\begin{array}{c} E\left[UV\right]=\mathbb{P}\left\{U=V\right\}-\mathbb{P}\left\{U\neq V\right\}\\ =2\mathbb{P}\left\{U=V\right\}-1\\ =2\mathbb{P}\left\{A=B\right\}-1 \end{array}$$

To translate, ℙ{U=V} is the probability that the alleles from our two focal samples agree with each other, while ℙ{U≠V} is the probability that they disagree. This implies that $E\left[UV\right]=1-2\pi_{ij},$ where $\pi_{ij}$ is the probability that two randomly chosen alleles differ, which is the genetic divergence.

Now, here is a generative model that gives us the form of the covariance we have postulated. To decide whether or not *A* and *B* will agree, first each sample randomly chooses a layer: call these layers *I* and *J*. The probability that *A* chooses layer *k* is $\mathbb{P}(I=k)=w_{i}^{\left(k\right)},$ the *i*th sample’s admixture proportion in the *k*th layer. The same holds true for *B*. If they do not choose the same layer, the probability that they agree is $p_{\gamma }.$ If they do choose the same layer, then they agree with a probability $1/2+p_{\gamma }+q_{ij}^{\left(k\right)}$ that depends on their distance apart. By the above, the probability of agreement is ℙ{A=B}=2cov[A,B]+1/2, and so we can define

$$\begin{array}{l} p_{\gamma }=2\left(\gamma +\delta_{ij}\eta_{i}\right)\\ q_{ij}^{\left(k\right)}=2\alpha_{0}^{\left(k\right)}\exp\left(-{\left(\alpha_{D}^{\left(k\right)}D_{ij}\right)}^{\alpha_{2}^{\left(k\right)}}\right)+2\varphi^{\left(k\right)}. \end{array}$$

One way to summarize the contribution of each layer is to partition the probability of agreement into contributions due to agreement “in” each layer. So, the contribution from layer *k* to agreement between *i* and *j* is

$$\frac{w_{i}^{\left(k\right)}w_{j}^{\left(k\right)}\left(1/2+q_{ij}^{\left(k\right)}\right)}{\left(1-\sum_{k=1}^{K}w_{i}^{\left(k\right)}w_{j}^{\left(k\right)}\right)\left(1/2+p_{\gamma }\right)+\sum_{k=1}^{K}w_{i}^{\left(k\right)}w_{j}^{\left(k\right)}\left(1/2+q_{ij}^{\left(k\right)}\right),}$$

which is the probability, given that they agree, that they agree thanks to layer *k*. Because our signal comes from *variation* in covariance, we omit the $p_{\gamma }$ terms (*i.e.*, we condition on agreement not due to “background” levels of agreement in the interpretations above). Stated in this way, this quantity is the relative contribution of the $k^{\text{th}}$ layer to the (model-based) kinship coefficient between *i* and *j*.

This suggests defining the overall contribution of layer *k* to agreement, $\xi^{\left(k\right)}$, to be the average of that quantity over *i* and *j*:

$$\xi^{\left(k\right)}=\sum_{i=1}^{N}\sum_{j=i}^{N}w_{i}^{\left(k\right)}w_{j}^{\left(k\right)}\left(2\alpha_{0}^{\left(k\right)}\left(\exp\left(-{\left(\alpha_{D}^{\left(k\right)}D_{i,j}\right)}^{\alpha_{2}^{\left(k\right)}}\right)\right)+2\varphi^{\left(k\right)}+\frac{1}{2}\right),$$ (16)

which is that layer’s contribution to agreement between samples summed over the upper triangle (excluding the diagonal) of the covariance matrix. We define the contribution of the *k*th layer, ${\text{Ξ}}^{\left(k\right)},$ as the relative contribution of the *k*th layer to total agreement:

$${\text{Ξ}}^{\left(k\right)}=\frac{\xi^{\left(k\right)}}{\sum_{k=1}^{K}\xi^{\left(k\right)}}.$$ (17)

This is the quantity that is plotted in Figure 4 and Figure 9.

### Simulation Details

We wished to simulate data under a model that had some biological realism, but at the same time had unambiguous true admixture proportions (so as to test the behavior of the method). This second requirement precluded scenarios of, *e.g.*, recent secondary contact between populations expanding out of different refugia, which would have more biological realism, but no unambiguous ancestry proportions for admixed populations. Here, we describe in more detail the procedure we use to simulate our test dataset, using a cartoon schematic with K=2 as an example (Figure A1).

Using the program ms (Hudson 2002), we generated discrete population structure by simulating *K* distinct populations, each of which split from a common ancestor $\tau_{\text{s}}$ units of coalescent time in the past, without subsequent migration between them. Then, to generate continuous differentiation within each population, at time $\tau_{\text{e}}$ in the past, each of these discrete populations instantaneously colonizes an independent lattice of demes, for which we use a stepping stone model with symmetric migration to nearest neighbors (eight neighbors, including diagonals).

Finally, at time $\tau_{\text{a}}$ in the past we generate a single dataset by collapsing those *K* discrete lattices into a single grid of demes that are admixed to various degrees from these different layers. We wish to simulate realistic patterns of admixture (and thereby set a more difficult test for the method), by generating spatially autocorrelated admixture proportions in each diverged population. To do so, we first place *K* equidistant points on the circle centered on our lattice. These points serve as “foci” of ancestry in each of the *K* layers. We then calculate the distance from each deme in the sampled lattice to each of these *K* foci, and draw admixture proportions for each deme from a Dirichlet distribution for which the concentration parameter for deme *i* in layer *k* is inversely proportional to the distance between deme *i* and focus *k*. This creates a pattern in which the admixture proportions in a given layer decreases with the distance from that layer’s focus, as might be expected if a spatial process were mediating admixture between diverged populations.

The parameters used to simulate the data were as follows: a diploid population size of 1000, a migration rate between neighboring demes of 0.4, a deep split time between layers of 500 (corresponding to $\tau_{\text{s}}$ in Figure A1), an expansion event across layers of 250 (corresponding to $\tau_{\text{e}}$ in Figure A1), and an admixture event between layers in the immediate past ($1\times 10^{-4}$). The times and rates reported above have already been scaled by 4N (as per ms syntax), and therefore give the values fed directly to ms. We used the -s option to sample a single segregating site per coalescent history, and simulated $1\times 10^{4}$ independent histories — corresponding to the same number of independent loci — in each dataset, with 10 diploid genotypes generated per deme at each locus.

## References

[bib1] Alexander D. H. Lange K., 2011 Enhancements to the admixture algorithm for individual ancestry estimation. BMC Bioinformatics 12: 246 10.1186/1471-2105-12-246 21682921 PMC3146885

[bib2] Alexander D. H. Novembre J. Lange K., 2009 Fast model-based estimation of ancestry in unrelated individuals. Genome Res. 19: 1655–1664. 10.1101/gr.094052.109 19648217 PMC2752134

[bib3] Barton N. H., 2008 The effect of a barrier to gene flow on patterns of geographic variation. Genet. Res. 90: 139–149. 10.1017/S0016672307009081 18289408

[bib4] Barton N. H. Depaulis F. Etheridge A. M., 2002 Neutral evolution in spatially continuous populations. Theor. Popul. Biol. 61: 31–48. 10.1006/tpbi.2001.1557 11895381

[bib5] Barton N. H. Etheridge A. M. Véber A., 2013 Modelling evolution in a spatial continuum. J. Stat. Mech. 2013: P01002 10.1088/1742-5468/2013/01/P01002

[bib6] Bradburd, G. S., P. L. Ralph, and G. M. Coop, 2013 Disentangling the effects of geographic and ecological isolation on genetic differentiation. Evolution 67: 3258–3273 10.1111/evo.12193 PMC3808528 24102455

[bib7] Bradburd, G. S., P. L. Ralph, and G. M. Coop, 2016 A spatial framework for understanding population structure and admixture. PLoS Genet. 12: e1005703 10.1371/journal.pgen.1005703 PMC4714911 26771578

[bib8] Byun S. A. Koop B. F. Reimchen T. E., 1997 North American black bear mtDNA phylogeography: implications for morphology and the haida gwaii glacial refugium controversy. Evolution 51: 1647–1653. 28568643 10.1111/j.1558-5646.1997.tb01487.x

[bib9] Carpenter B. Gelman A. Hoffman M. D. Lee D. Goodrich B. 2017 Stan: a probabilistic programming language. J. Stat. Softw. 76: 10.18637/jss.v076.i01 PMC9788645 36568334

[bib10] Cavalli-Sforza L. L. Piazza A., 1975 Analysis of evolution: evolutionary rates, independence and treeness. Theor. Popul. Biol. 8: 127–165. 10.1016/0040-5809(75)90029-5 1198349

[bib11] Caye K. Jay F. Michel O. François O., 2018 Fast inference of individual admixture coefficients using geographic data. Ann. Appl. Stat. 12: 586–608. 10.1214/17-AOAS1106

[bib12] Corander J. Waldmann P. Sillanpää M. J., 2003 Bayesian analysis of genetic differentiation between populations. Genetics 163: 367–374. 12586722 10.1093/genetics/163.1.367 PMC1462429

[bib13] Cronk Q. C. B., 2005 Plant eco-devo: the potential of poplar as a model organism. New Phytol. 166: 39–48. 10.1111/j.1469-8137.2005.01369.x 15760349

[bib14] Diggle P. J. Tawn J. A. Moyeed R. A., 1998 Model-based geostatistics. J. Roy. Stat. Soc. C. Appl. Stat. 47: 299–350.

[bib15] Eckenwalder J. E., 1984 Natural intersectional hybridization between North American species of Populus (salicaceae) in sections Aigeiros and Tacamahaca. ii. Taxonomy. Can. J. Bot. 62: 325–335. 10.1139/b84-051

[bib16] Engelhardt B. E. Stephens M., 2010 Analysis of population structure: a unifying framework and novel methods based on sparse factor analysis. PLoS Genet. 6: e1001117 10.1371/journal.pgen.1001117 20862358 PMC2940725

[bib17] Epperson B., 2003 *Geographical Genetics. Monographs in Population Biology*. Princeton University Press, Princeton, NJ.

[bib18] Evanno G. Regnaut S. Goudet J., 2005 Detecting the number of clusters of individuals using the software structure: a simulation study. Mol. Ecol. 14: 2611–2620. 10.1111/j.1365-294X.2005.02553.x 15969739

[bib19] Falush D. Stephens M. Pritchard J. K., 2003 Inference of population structure using multilocus genotype data: linked loci and correlated allele frequencies. Genetics 164: 1567–1587. 12930761 10.1093/genetics/164.4.1567 PMC1462648

[bib20] Falush D. van Dorp L. Lawson D., 2016 A tutorial on how (not) to over-interpret STRUCTURE/ADMIXTURE bar plots. bioRxiv 066431; 10.1101/066431. PMC6092366 30108219

[bib21] Frantz A. C. Cellina S. Krier A. Schley L. Burke T., 2009 Using spatial Bayesian methods to determine the genetic structure of a continuously distributed population: clusters or isolation by distance? J. Appl. Ecol. 46: 493–505. 10.1111/j.1365-2664.2008.01606.x

[bib22] Geraldes A. DiFazio S. P. Slavov G. T. Ranjan P. Muchero W. 2013 A 34k SNP genotyping array for Populus trichocarpa: design, application to the study of natural populations and transferability to other populus species. Mol. Ecol. Resour. 13: 306–323. 10.1111/1755-0998.12056 23311503

[bib23] Geraldes A. Farzaneh N. Grassa C. J. McKown A. D. Guy R. D. 2014 Landscape genomics of Populus trichocarpa: the role of hybridization, limited gene flow, and natural selection in shaping patterns of population structure. Evolution 68: 3260–3280. 10.1111/evo.12497 25065449

[bib24] Guillot G. Mortier F. Estoup A., 2005 Geneland: a computer package for landscape genetics. Mol. Ecol. Notes 5: 712–715. 10.1111/j.1471-8286.2005.01031.x

[bib25] Haak W. Lazaridis I. Patterson N. Rohland N. Mallick S. 2015 Massive migration from the steppe was a source for Indo-European languages in Europe. Nature 522: 207–211. 10.1038/nature14317 25731166 PMC5048219

[bib26] Hoffman M. D. Gelman A., 2014 The No-U-Turn sampler: adaptively setting path lengths in Hamiltonian Monte Carlo. J. Mach. Learn. Res. arXiv:1111.4246v1

[bib27] Hubisz M. J. Falush D. Stephens M. Pritchard J. K., 2009 Inferring weak population structure with the assistance of sample group information. Mol. Ecol. Resour. 9: 1322–1332. 10.1111/j.1755-0998.2009.02591.x 21564903 PMC3518025

[bib28] Hudson R. R., 2002 Generating samples under a Wright–Fisher neutral model of genetic variation. Bioinformatics 18: 337–338. 10.1093/bioinformatics/18.2.337 11847089

[bib29] Huelsenbeck J. P. Andolfatto P., 2007 Inference of population structure under a Dirichlet process model. Genetics 175: 1787–1802. 10.1534/genetics.106.061317 17237522 PMC1855109

[bib30] Irwin D. E. Bensch S. Price T. D., 2001 Speciation in a ring. Nature 409: 333–337. 10.1038/35053059 11201740

[bib31] Joseph T. A. Pe’er I., 2018 Inference of population structure from ancient DNA. bioRxiv 261131; 10.1101/261131.

[bib32] Keller S. R. Olson M. S. Silim S. Schroeder W. Tiffin P., 2010 Genomic diversity, population structure, and migration following rapid range expansion in the balsam poplar, Populus balsamifera. Mol. Ecol. 19: 1212–1226. 10.1111/j.1365-294X.2010.04546.x 20163548

[bib33] Kimura M. Weiss G. H., 1964 The stepping stone model of population structure and the decrease of genetic correlation with distance. Genetics 49: 561–576. 17248204 10.1093/genetics/49.4.561 PMC1210594

[bib34] Lawson D. J. Hellenthal G. Myers S. Falush D., 2012 Inference of population structure using dense haplotype data. PLoS Genet. 8: e1002453 10.1371/journal.pgen.1002453 22291602 PMC3266881

[bib35] Lazaridis I. Patterson N. Mittnik A. Renaud G. Mallick S. 2014 Ancient human genomes suggest three ancestral populations for present-day Europeans. Nature 513: 409–413. 10.1038/nature13673 25230663 PMC4170574

[bib36] Linck E. B. Battey C. J., 2017 Minor allele frequency thresholds strongly affect population structure inference with genomic datasets. bioRxiv. 188623; 10.1101/188623 30659755

[bib37] Malécot G., 1969 *The Mathematics of Heredity*. W. H. Freeman, San Francisco.

[bib38] McKown A. D. Guy R. D. Klapste J. Geraldes A. Friedmann M. 2014 Geographical and environmental gradients shape phenotypic trait variation and genetic structure in Populus trichocarpa. New Phytol. 201: 1263–1276. 10.1111/nph.12601 24491114

[bib39] McVean G., 2009 A genealogical interpretation of principal components analysis. PLoS Genet. 5: e1000686 10.1371/journal.pgen.1000686 19834557 PMC2757795

[bib40] Meirmans P., 2009 Genodive version 2.0 b14. Computer software distributed by the author. Accessed : May 12th, 2018. Available at: http://www. bentleydrummer.nl/software/software/GenoDive.html.

[bib41] Meirmans P. G., 2012 The trouble with isolation by distance. Mol. Ecol. 21: 2839–2846. 10.1111/j.1365-294X.2012.05578.x 22574758

[bib42] Menozzi P. Piazza A. Cavalli-Sforza L., 1978 Synthetic maps of human gene frequencies in Europeans. Science 201: 786–792. 10.1126/science.356262 356262

[bib43] Moritz C., 1994 Defining “evolutionarily significant units” for conservation. Trends Ecol. Evol. 9: 373–375. 10.1016/0169-5347(94)90057-4 21236896

[bib44] Moritz C. Funk V. Sakai A. K., 2002 Strategies to protect biological diversity and the evolutionary processes that sustain it. Syst. Biol. 51: 238–254. 10.1080/10635150252899752 12028731

[bib45] Nagylaki T., 1978 A diffusion model for geographically structured populations. J. Math. Biol. 6: 375–382. 10.1007/BF02463002 7365330

[bib46] Nagylaki T. Barcilon V., 1988 The influence of spatial inhomogeneities of neutral models of geographical variation. II. The semi-infinite linear habitat. Theor. Popul. Biol. 33: 311–343. 10.1016/0040-5809(88)90018-4

[bib47] Nielsen R. Akey J. M. Jakobsson M. Pritchard J. K. Tishkoff S., 2017 Tracing the peopling of the world through genomics. Nature 541: 302–310. 10.1038/nature21347 28102248 PMC5772775

[bib48] Novembre J. Stephens M., 2008 Interpreting principal component analyses of spatial population genetic variation. Nat. Genet. 40: 646–649. 10.1038/ng.139 18425127 PMC3989108

[bib49] Patterson N. Price A. L. Reich D., 2006 Population structure and eigenanalysis. PLoS Genet. 2: e190 10.1371/journal.pgen.0020190 17194218 PMC1713260

[bib50] Patterson N. Moorjani P. Luo Y. Mallick S. Rohland N., 2012 Ancient admixture in human history. Genetics 192: 1065–1093. 10.1534/genetics.112.145037 22960212 PMC3522152

[bib51] Peter B. M., 2016 Admixture, population structure and f-statistics. Genetics 202: 1485–1501. 10.1534/genetics.115.183913 26857625 PMC4905545

[bib52] Petkova D. Novembre J. Stephens M., 2016 Visualizing spatial population structure with estimated effective migration surfaces. Nat. Genet. 48: 94–100. 10.1038/ng.3464 26642242 PMC4696895

[bib53] Picard R. R. Cook R. D., 1984 Cross-validation of regression models. J. Am. Stat. Assoc. 79: 575–583. 10.1080/01621459.1984.10478083

[bib54] Pickrell J. K. Pritchard J. K., 2012 Inference of population splits and mixtures from genome-wide allele frequency data. PLoS Genet. 8: e1002967 10.1371/journal.pgen.1002967 23166502 PMC3499260

[bib55] Price A. L. Patterson N. J. Plenge R. M. Weinblatt M. E. Shadick N. A., 2006 Principal components analysis corrects for stratification in genome-wide association studies. Nat. Genet. 38: 904–909. 10.1038/ng1847 16862161

[bib56] Pritchard J. K. Stephens M. Donnelly P., 2000 Inference of population structure using multilocus genotype data. Genetics 155: 945–959. 10835412 10.1093/genetics/155.2.945 PMC1461096

[bib57] Puckett E. E. Etter P. D. Johnson E. A. Eggert L. S., 2015 Phylogeographic analyses of American black bears (Ursus americanus) suggest four glacial refugia and complex patterns of postglacial admixture. Mol. Biol. Evol. 32: 2338–2350. 10.1093/molbev/msv114 25989983

[bib58] Raj A. Stephens M. Pritchard J. K., 2014 fastSTRUCTURE: variational inference of population structure in large SNP data sets. Genetics 197: 573–589. 10.1534/genetics.114.164350 24700103 PMC4063916

[bib59] Ringbauer H. Kolesnikov A. Field D. L. Barton N. H., 2018 Estimating barriers to gene flow from distorted isolation-by-distance patterns. Genetics 208: 1231–1245. 10.1534/genetics.117.300638 29311149 PMC5844333

[bib60] Rosenberg N. A. Mahajan S. Ramachandran S. Zhao C. Pritchard J. K., 2005 Clines, clusters, and the effect of study design on the inference of human population structure. PLoS Genet. 1: e70. 16355252 10.1371/journal.pgen.0010070 PMC1310579

[bib61] Sawyer S., 1976 Results for the stepping stone model for migration in population genetics. Ann. Probab. 4: 699–728. 10.1214/aop/1176995980

[bib62] Schraiber J., 2017 Assessing the relationship of ancient and modern populations. bioRxiv. 113779; 10.1101/113779 PMC5753871 29167200

[bib63] Serre D. Pääbo S., 2004 Evidence for gradients of human genetic diversity within and among continents. Genome Res. 14: 1679–1685. 10.1101/gr.2529604 15342553 PMC515312

[bib64] Sexton J. P. Hangartner S. B. Hoffmann A. A., 2014 Genetic isolation by environment or distance: which pattern of gene flow is most common? Evolution 68: 1–15. 10.1111/evo.12258 24111567

[bib66] Slatkin M., 1985 Gene flow in natural populations. Annu. Rev. Ecol. Syst. 16: 393–430. 10.1146/annurev.es.16.110185.002141

[bib67] Slatkin M. Racimo F., 2016 Ancient DNA and human history. Proc. Natl. Acad. Sci. USA 113: 6380–6387. 10.1073/pnas.1524306113 27274045 PMC4988579

[bib68] Slavov G. T. DiFazio S. P. Martin J. Schackwitz W. Muchero W. 2012 Genome resequencing reveals multiscale geographic structure and extensive linkage disequilibrium in the forest tree Populus trichocarpa. New Phytol. 196: 713–725. 10.1111/j.1469-8137.2012.04258.x 22861491

[bib69] Stan Development Team, 2015 Stan: A C++ library for probability and sampling, version 2.10.0. http://mc-stan.org

[bib70] Stan Development Team, 2016 RStan: the R interface to Stan, version 2.10.1. http://mc-stan.org

[bib71] Stone K. D. Cook J. A., 2000 Phylogeography of black bears (Ursus americanus) of the pacific northwest. Can. J. Zool. 78: 1218–1223. 10.1139/z00-042

[bib72] Suarez-Gonzalez A. Hefer C. A. Christe C. Corea O. Lexer C. 2016 Genomic and functional approaches reveal a case of adaptive introgression from Populus balsamifera (balsam poplar) in P. trichocarpa (black cottonwood). Mol. Ecol. 25: 2427–2442. 10.1111/mec.13539 26825293

[bib73] Verity R. Nichols R. A., 2016 Estimating the number of subpopulations (K) in structured populations. Genetics 203: 1827–1839. 10.1534/genetics.115.180992 27317680 PMC4981280

[bib74] Wake D. B. Schneider C. J., 1998 Taxonomy of the plethodontid salamander genus ensatina. Herpetologica 54: 279–298.

[bib75] Waples R., 1998 Separating the wheat from the chaff: patterns of genetic differentiation in high gene flow species. J. Hered. 89: 438–450. 10.1093/jhered/89.5.438

[bib76] Wasser S. K. Shedlock A. M. Comstock K. Ostrander E. Mutayoba B. 2004 Assigning African elephant DNA to geographic region of origin: applications to the ivory trade. Proc. Natl. Acad. Sci. USA 101: 14847–14852. 10.1073/pnas.0403170101 15459317 PMC522003

[bib77] Wooding S. Ward R., 1997 Phylogeography and pleistocene evolution in the North American black bear. Mol. Biol. Evol. 14: 1096–1105. 10.1093/oxfordjournals.molbev.a025719 9364767

[bib78] Wright S., 1943 Isolation by distance. Genetics 28: 114–138. 17247074 10.1093/genetics/28.2.114 PMC1209196

[bib79] Wright S., 1949 The genetical structure of populations. Ann. Eugen. 15: 323–354. 10.1111/j.1469-1809.1949.tb02451.x 24540312

[bib80] Xie C.-Y. Ying C. C. Yanchuk A. D. Holowachuk D. L., 2009 Ecotypic mode of regional differentiation caused by restricted gene migration: a case in black cottonwood (Populus trichocarpa) along the pacific northwest coast. Can. J. For. Res. 39: 519–525. 10.1139/X08-190

[bib81] Xie C. Y. Carlson M. R. Ying C. C., 2012 Ecotypic mode of regional differentiation of black cottonwood (Populus trichocarpa) due to restricted gene migration: further evidence from a field test on the northern coast of British Columbia. Can. J. For. Res. 42: 400–405. 10.1139/x11-187

[bib82] Zhou L. Holliday J. A., 2012 Targeted enrichment of the black cottonwood (Populus trichocarpa) gene space using sequence capture. BMC Genomics 13: 703 10.1186/1471-2164-13-703 23241106 PMC3542275

